# Bis[2-(5-methyl­sulfanyl-1,3,4-oxadiazol-2-yl-κ*N* ^3^)phenolato-κ*O* ^1^]copper(II)

**DOI:** 10.1107/S1600536812026815

**Published:** 2012-06-20

**Authors:** Souheila Ouilia, Chahrazed Beghidja, Adel Beghidja, François Michaud

**Affiliations:** a Unité de Recherche de Chimie de l’Environnement et Moléculaire Structurale (CHEMS), Faculté des Sciences Exactes, Département de Chimie, Université Mentouri, 25000 Constantine, Algeria; b Centre de Diffractométrie X, Université de Bretagne Occidentale, BP 809, 29285 Brest Cedex, France

## Abstract

In the title complex, [Cu(C_9_H_7_N_2_O_2_S)_2_], the Cu^II^ ion, located on an inversion center, adopts an N_2_O_2_ square-planar coord­ination. The 2-(5-methyl­sulfanyl-1,3,4-oxadiazol-2-yl)phenolate ligand is chelated to the central Cu^II^ ion in an *N*,*O*-bidentate manner.

## Related literature

For general background to derivatives of dithio­carbazate ligands and their metal complexes, see: Beghidja *et al.* (2005 ▶; 2006 ▶); Bouchameni *et al.* (2011 ▶); Beghidja, Bouslimani & Welter (2007 ▶); Beghidja, Rogez & Welter (2007 ▶). For similar structures, see: Kala *et al.* (2007 ▶); Liu *et al.* (2008 ▶); Zhang *et al.* (2001 ▶). For the preparation of the ligand, see: Dolman *et al.* (2006 ▶); Young & Wood (1955 ▶).
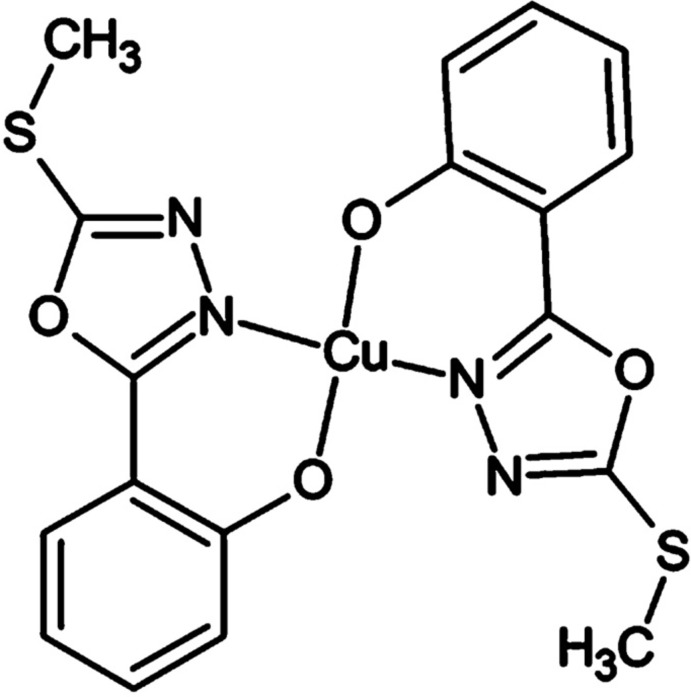


## Experimental

### 

#### Crystal data

[Cu(C_9_H_7_N_2_O_2_S)_2_]
*M* *_r_* = 478.02
Monoclinic, 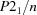

*a* = 12.5695 (7) Å
*b* = 4.4216 (3) Å
*c* = 17.3861 (9) Å
β = 106.005 (6)°
*V* = 928.81 (9) Å^3^
*Z* = 2
Mo *K*α radiation
μ = 1.44 mm^−1^
*T* = 170 K
0.18 × 0.12 × 0.09 mm

#### Data collection

Oxford Diffraction Xcalibur CCD diffractometer
Absorption correction: multi-scan (*CrysAlis RED*; Oxford Diffraction, 2007 ▶) *T*_min_ = 0.926, *T*_max_ = 1.000
6693 measured reflections
1906 independent reflections
1250 reflections with *I* > 2σ(*I*)
*R*_int_ = 0.037

#### Refinement

*R*[*F* ^2^ > 2σ(*F* ^2^)] = 0.033
*wR*(*F* ^2^) = 0.066
*S* = 0.99
1906 reflections
133 parameters
H-atom parameters constrained
Δρ_max_ = 0.31 e Å^−3^
Δρ_min_ = −0.18 e Å^−3^

### 

Data collection: *CrysAlis CCD* (Oxford Diffraction, 2007 ▶); cell refinement: *CrysAlis CCD*; data reduction: *CrysAlis RED* (Oxford Diffraction, 2007 ▶); program(s) used to solve structure: *SIR97* (Altomare *et al.*, 1999 ▶); program(s) used to refine structure: *SHELXL97* (Sheldrick, 2008 ▶); molecular graphics: *ATOMS* (Dowty, 1995 ▶); software used to prepare material for publication: *WinGX* (Farrugia, 1999 ▶) and *PLATON* (Spek, 2009 ▶).

## Supplementary Material

Crystal structure: contains datablock(s) global, I. DOI: 10.1107/S1600536812026815/hp2039sup1.cif

Structure factors: contains datablock(s) I. DOI: 10.1107/S1600536812026815/hp2039Isup2.hkl

Additional supplementary materials:  crystallographic information; 3D view; checkCIF report

## Figures and Tables

**Table 1:** Selected bond lengths (Å)

Cu1—O2	1.896 (2)
Cu1—N1	1.9746 (19)

**Fig. 1.**
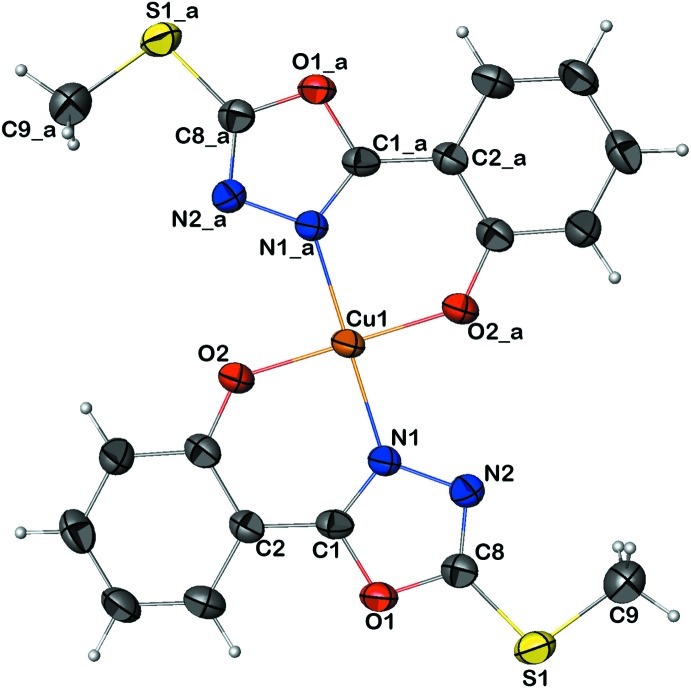
The molecular structure of (I), with atom labels and 50% probability displacement ellipsoids for non-H atoms.

**Fig. 2.**
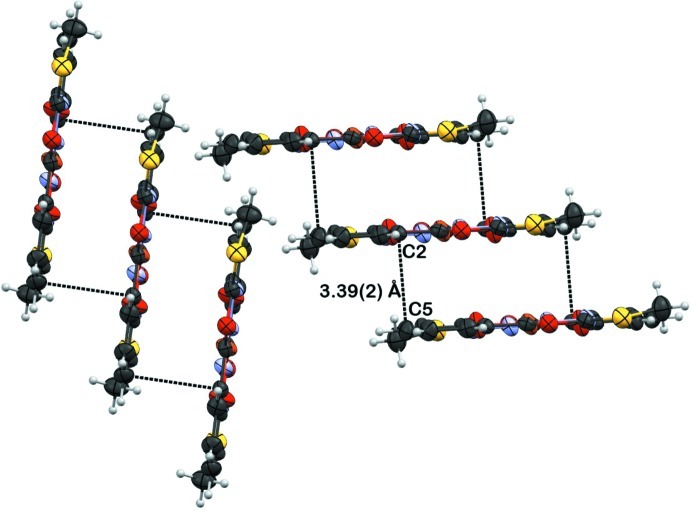
Linking of the layers in the structure via van der Waals interactions.

## supplementary crystallographic information

### Comment

The molecular structure of the complex (1) shows that the Cu^II^ ion is located on an inversion center and chelated by two bidentate anions H*L*^-^ (Fig. 1). This ligand has been obtained from the *in situ* cyclization of 2-hydroxy [bis(methylsulfanyl)methylene]hydrazide H*L*^(1)^ described previously by (Young *et al.*,1955; Dolman *et al.*, 2006). The title mononuclear complex, [Cu (C_9_H_7_O_2_N_2_S)_2_] (1) has a square-plane geometry formed by the N_2_O_2_ donor atoms (N1, O2). Several mononuclear compounds with similar structures have been reported previously (Kala *et al.*, 2007; Liu *et al.*, 2008). The whole molecule is planar with a small deviation at C8 from the mean plane. The distances in the coordination planes around the Cu^II^ ion [Cu_1_—N_1_= 1.975 (19) Å and Cu_1_—O_2_= 1.896 (2) Å] are in agreement with other square-planar complexes, such as [Cu(C_15_H_22_O)2] [Cu—O = 1.88 (3) Å and Cu—N = 2.00 (3) Å; (Zhang *et al.*, 2001)]. From a supramolecular point of view, this structure can be described as a zigzag chain within which the molecular complexes are connected to each other *via* the weak hydrogen bonding C—H···O. In the crystal the layers are held together by normal van der Waals interactions (Fig. 2).

### Experimental

The ligand H*L*^(1)^ (0.128 g, 0.05 mmol) was dissolved in minimum of DMF. The solution of CuCl_2_.2H_2_O (0.0085 g, 0.05 mmol) in DMF was added to the first when the ligand was dissolved completely. Green crystals of the complex 1 were isolated from the solution after two weeks.

### Refinement

All H atoms were placed at calculated positions and treated as riding on their parent atoms with C—H = 0.93–0.96 Å, and *U*_iso_ (H) = 1.5Ueq(C) for methyl H atoms and 1.2Ueq(C) for the others.

### Crystal data

**Table:**

[Cu(C_9_H_7_N_2_O_2_S)_2_]	*F*(000) = 486
*M**_r_* = 478.02	Least Squares Treatment of 25 SET4 setting angles.
Monoclinic, *P*2_1_/*n*	*D*_x_ = 1.709 Mg m^−^^3^
Hall symbol: -P 2yn	Mo *K*α radiation, λ = 0.71073 Å
*a* = 12.5695 (7) Å	Cell parameters from 2354 reflections
*b* = 4.4216 (3) Å	θ = 3.3–31.6°
*c* = 17.3861 (9) Å	µ = 1.44 mm^−^^1^
β = 106.005 (6)°	*T* = 170 K
*V* = 928.81 (9) Å^3^	Plates, green
*Z* = 2	0.18 × 0.12 × 0.09 mm

### Data collection

**Table:**

Oxford Diffraction Xcalibur CCD diffractometer	1906 independent reflections
Radiation source: Enhance (Mo) X-ray Source	1250 reflections with *I* > 2σ(*I*)
Graphite monochromator	*R*_int_ = 0.037
Detector resolution: 18.4 pixels mm^-1^	θ_max_ = 26.4°, θ_min_ = 3.4°
ω and φ scans	*h* = −15→15
Absorption correction: multi-scan (*CrysAlis RED*; Oxford Diffraction, 2007)	*k* = −5→5
*T*_min_ = 0.926, *T*_max_ = 1.000	*l* = −21→14
6693 measured reflections	

### Refinement

**Table:**

Refinement on *F*^2^	Primary atom site location: structure-invariant direct methods
Least-squares matrix: full	Secondary atom site location: difference Fourier map
*R*[*F*^2^ > 2σ(*F*^2^)] = 0.033	Hydrogen site location: inferred from neighbouring sites
*wR*(*F*^2^) = 0.066	H-atom parameters constrained
*S* = 0.99	*w* = 1/[σ^2^(*F*_o_^2^) + (0.0283*P*)^2^] where *P* = (*F*_o_^2^ + 2*F*_c_^2^)/3
1906 reflections	(Δ/σ)_max_ = 0.004
133 parameters	Δρ_max_ = 0.31 e Å^−^^3^
0 restraints	Δρ_min_ = −0.18 e Å^−^^3^

### Special details

**Table:**

Geometry. Bond distances, angles *etc*. have been calculated using the rounded fractional coordinates. All su's are estimated from the variances of the (full) variance-covariance matrix. The cell e.s.d.'s are taken into account in the estimation of distances, angles and torsion angles
Refinement. Refinement on *F*^2^ for ALL reflections except those flagged by the user for potential systematic errors. Weighted *R*-factors *wR* and all goodnesses of fit *S* are based on *F*^2^, conventional *R*-factors *R* are based on *F*, with *F* set to zero for negative *F*^2^. The observed criterion of *F*^2^ > σ(*F*^2^) is used only for calculating -*R*-factor-obs *etc*. and is not relevant to the choice of reflections for refinement. *R*-factors based on *F*^2^ are statistically about twice as large as those based on *F*, and *R*-factors based on ALL data will be even larger.

### Fractional atomic coordinates and isotropic or equivalent isotropic displacement parameters (Å^2^)

**Table:**

	*x*	*y*	*z*	*U*_iso_*/*U*_eq_	
Cu1	0.50000	0.00000	1.00000	0.0381 (1)	
S1	0.45296 (6)	0.47186 (17)	0.68566 (4)	0.0455 (3)	
O1	0.58495 (13)	0.1114 (4)	0.79047 (9)	0.0362 (6)	
O2	0.62190 (14)	−0.2686 (5)	1.01766 (9)	0.0506 (7)	
N1	0.51554 (16)	0.0936 (5)	0.89257 (11)	0.0347 (7)	
N2	0.44837 (16)	0.2890 (5)	0.83525 (11)	0.0387 (8)	
C1	0.59434 (18)	−0.0065 (6)	0.86434 (13)	0.0315 (7)	
C2	0.68113 (19)	−0.2153 (6)	0.89831 (14)	0.0326 (8)	
C3	0.68883 (19)	−0.3359 (6)	0.97479 (15)	0.0356 (8)	
C4	0.7755 (2)	−0.5413 (6)	1.00571 (15)	0.0424 (9)	
C5	0.8503 (2)	−0.6167 (6)	0.96422 (17)	0.0474 (10)	
C6	0.8424 (2)	−0.4940 (7)	0.88955 (16)	0.0455 (9)	
C7	0.7584 (2)	−0.2969 (6)	0.85724 (16)	0.0429 (10)	
C8	0.4933 (2)	0.2903 (6)	0.77720 (14)	0.0346 (8)	
C9	0.3251 (2)	0.6276 (7)	0.69352 (17)	0.0602 (11)	
H4	0.78230	−0.62840	1.05550	0.0510*	
H5	0.90710	−0.75200	0.98660	0.0570*	
H6	0.89350	−0.54500	0.86190	0.0550*	
H7	0.75220	−0.21510	0.80690	0.0510*	
H9A	0.29080	0.74020	0.64590	0.0900*	
H9B	0.33910	0.75940	0.73910	0.0900*	
H9C	0.27680	0.46690	0.69980	0.0900*	

### Atomic displacement parameters (Å^2^)

**Table:**

	*U*^11^	*U*^22^	*U*^33^	*U*^12^	*U*^13^	*U*^23^
Cu1	0.0309 (2)	0.0596 (3)	0.0265 (2)	0.0096 (2)	0.0123 (2)	0.0020 (3)
S1	0.0469 (4)	0.0576 (5)	0.0340 (4)	−0.0015 (4)	0.0146 (3)	0.0092 (4)
O1	0.0361 (10)	0.0457 (11)	0.0307 (9)	−0.0003 (8)	0.0158 (8)	0.0006 (8)
O2	0.0442 (11)	0.0785 (14)	0.0346 (10)	0.0221 (10)	0.0200 (9)	0.0092 (11)
N1	0.0300 (11)	0.0482 (14)	0.0274 (11)	0.0054 (10)	0.0104 (9)	0.0020 (10)
N2	0.0340 (12)	0.0521 (15)	0.0303 (12)	0.0051 (11)	0.0093 (10)	0.0039 (11)
C1	0.0301 (12)	0.0398 (14)	0.0261 (12)	−0.0068 (14)	0.0102 (10)	−0.0058 (14)
C2	0.0285 (13)	0.0385 (15)	0.0316 (13)	−0.0021 (11)	0.0098 (11)	−0.0059 (12)
C3	0.0289 (13)	0.0443 (16)	0.0341 (14)	−0.0017 (12)	0.0095 (11)	−0.0084 (13)
C4	0.0397 (15)	0.0520 (19)	0.0342 (13)	0.0079 (13)	0.0078 (12)	0.0011 (14)
C5	0.0398 (16)	0.0462 (17)	0.0562 (19)	0.0112 (13)	0.0135 (14)	−0.0070 (15)
C6	0.0398 (14)	0.0508 (17)	0.0535 (16)	0.0065 (15)	0.0256 (13)	−0.0016 (17)
C7	0.0426 (16)	0.0489 (18)	0.0438 (16)	0.0004 (14)	0.0230 (13)	−0.0031 (14)
C8	0.0320 (14)	0.0401 (16)	0.0315 (14)	−0.0036 (12)	0.0086 (12)	−0.0033 (12)
C9	0.060 (2)	0.069 (2)	0.0527 (19)	0.0110 (16)	0.0175 (16)	0.0157 (16)

### Geometric parameters (Å, º)

**Table:**

Cu1—O2	1.896 (2)	C2—C3	1.411 (3)
Cu1—N1	1.9746 (19)	C2—C7	1.402 (4)
Cu1—O2^i^	1.896 (2)	C3—C4	1.406 (4)
Cu1—N1^i^	1.9746 (19)	C4—C5	1.375 (4)
S1—C8	1.729 (3)	C5—C6	1.385 (4)
S1—C9	1.788 (3)	C6—C7	1.365 (4)
O1—C1	1.361 (3)	C4—H4	0.9300
O1—C8	1.364 (3)	C5—H5	0.9300
O2—C3	1.303 (3)	C6—H6	0.9300
N1—N2	1.410 (3)	C7—H7	0.9300
N1—C1	1.298 (3)	C9—H9A	0.9600
N2—C8	1.285 (3)	C9—H9B	0.9600
C1—C2	1.427 (4)	C9—H9C	0.9600
			
Cu1···O2^ii^	3.555 (2)	C3···Cu1^vi^	3.876 (3)
Cu1···C3^ii^	3.876 (3)	C3···N1^vi^	3.381 (3)
Cu1···C4^ii^	3.991 (3)	C3···C1^vi^	3.554 (4)
Cu1···O2^iii^	3.555 (2)	C3···Cu1^iii^	3.876 (3)
Cu1···C3^iii^	3.876 (3)	C4···C2^vi^	3.543 (4)
Cu1···C4^iii^	3.991 (3)	C4···Cu1^vi^	3.991 (3)
S1···H6^iv^	3.1400	C4···C1^vi^	3.517 (4)
S1···H4^v^	3.0600	C4···Cu1^iii^	3.991 (3)
O1···N2	2.215 (3)	C5···C7^vi^	3.557 (4)
O1···C7^ii^	3.399 (3)	C5···C2^vi^	3.391 (4)
O2···N2^i^	2.928 (3)	C7···O1^vi^	3.399 (3)
O2···C1	2.839 (3)	C7···C5^ii^	3.557 (4)
O2···Cu1^vi^	3.555 (2)	C8···C1^ii^	3.538 (4)
O2···N1	2.735 (3)	C8···C2^ii^	3.471 (4)
O2···Cu1^iii^	3.555 (2)	C9···N2^x^	3.410 (3)
O2···N1^i^	2.740 (3)	C3···H9A^viii^	2.9300
O1···H6^vii^	2.8200	C4···H9A^viii^	2.7400
O1···H7	2.5000	C8···H9B^vi^	3.0000
O2···H9A^viii^	2.6200	C9···H9C^x^	2.9400
N1···O1	2.185 (3)	H4···S1^xi^	3.0600
N1···O2	2.735 (3)	H4···H9A^viii^	2.3100
N1···C3	2.946 (3)	H6···S1^xii^	3.1400
N1···C3^ii^	3.381 (3)	H6···O1^xiii^	2.8200
N1···O2^i^	2.740 (3)	H7···O1	2.5000
N2···O1	2.215 (3)	H9A···O2^xiv^	2.6200
N2···O2^i^	2.928 (3)	H9A···C3^xiv^	2.9300
N2···C9^ix^	3.410 (3)	H9A···C4^xiv^	2.7400
N2···H9C	2.8300	H9A···H4^xiv^	2.3100
N2···H9B	2.7800	H9B···N2	2.7800
C1···C3^ii^	3.554 (4)	H9B···C8^ii^	3.0000
C1···C4^ii^	3.517 (4)	H9B···H9C^x^	2.2200
C1···C8^vi^	3.538 (4)	H9C···N2	2.8300
C2···C4^ii^	3.543 (4)	H9C···C9^ix^	2.9400
C2···C5^ii^	3.391 (4)	H9C···H9B^ix^	2.2200
C2···C8^vi^	3.471 (4)		
			
O2—Cu1—N1	89.90 (8)	C3—C4—C5	121.7 (2)
O2—Cu1—O2^i^	180.00	C4—C5—C6	121.0 (2)
O2—Cu1—N1^i^	90.11 (8)	C5—C6—C7	118.9 (2)
O2^i^—Cu1—N1	90.11 (8)	C2—C7—C6	121.4 (2)
N1—Cu1—N1^i^	180.00	S1—C8—O1	116.41 (17)
O2^i^—Cu1—N1^i^	89.90 (8)	S1—C8—N2	130.1 (2)
C8—S1—C9	98.63 (13)	O1—C8—N2	113.5 (2)
C1—O1—C8	103.37 (18)	C3—C4—H4	119.00
Cu1—O2—C3	132.15 (16)	C5—C4—H4	119.00
Cu1—N1—N2	126.93 (15)	C4—C5—H5	120.00
Cu1—N1—C1	124.81 (17)	C6—C5—H5	119.00
N2—N1—C1	108.19 (19)	C5—C6—H6	121.00
N1—N2—C8	104.5 (2)	C7—C6—H6	121.00
O1—C1—N1	110.5 (2)	C2—C7—H7	119.00
O1—C1—C2	119.7 (2)	C6—C7—H7	119.00
N1—C1—C2	129.8 (2)	S1—C9—H9A	109.00
C1—C2—C3	118.7 (2)	S1—C9—H9B	109.00
C1—C2—C7	120.9 (2)	S1—C9—H9C	109.00
C3—C2—C7	120.4 (2)	H9A—C9—H9B	109.00
O2—C3—C2	124.4 (2)	H9A—C9—H9C	110.00
O2—C3—C4	118.9 (2)	H9B—C9—H9C	109.00
C2—C3—C4	116.7 (2)		
			
N1—Cu1—O2—C3	3.7 (2)	Cu1—N1—C1—O1	−176.81 (15)
N1^i^—Cu1—O2—C3	−176.3 (2)	N1—N2—C8—O1	0.2 (3)
O2—Cu1—N1—N2	178.6 (2)	N1—N2—C8—S1	177.9 (2)
O2^i^—Cu1—N1—N2	−1.4 (2)	O1—C1—C2—C7	1.2 (4)
O2—Cu1—N1—C1	−4.9 (2)	N1—C1—C2—C7	179.4 (3)
O2^i^—Cu1—N1—C1	175.1 (2)	O1—C1—C2—C3	−179.6 (2)
C9—S1—C8—O1	173.0 (2)	N1—C1—C2—C3	−1.4 (4)
C9—S1—C8—N2	−4.7 (3)	C3—C2—C7—C6	0.2 (4)
C8—O1—C1—N1	−0.1 (3)	C1—C2—C3—C4	179.8 (2)
C1—O1—C8—N2	−0.1 (3)	C1—C2—C7—C6	179.3 (3)
C8—O1—C1—C2	178.4 (2)	C1—C2—C3—O2	−0.4 (4)
C1—O1—C8—S1	−178.12 (18)	C7—C2—C3—O2	178.8 (2)
Cu1—O2—C3—C2	−1.8 (4)	C7—C2—C3—C4	−1.0 (4)
Cu1—O2—C3—C4	177.95 (18)	O2—C3—C4—C5	−178.6 (2)
C1—N1—N2—C8	−0.3 (3)	C2—C3—C4—C5	1.2 (4)
Cu1—N1—N2—C8	176.71 (18)	C3—C4—C5—C6	−0.5 (4)
N2—N1—C1—C2	−178.1 (3)	C4—C5—C6—C7	−0.3 (4)
N2—N1—C1—O1	0.2 (3)	C5—C6—C7—C2	0.5 (4)
Cu1—N1—C1—C2	4.9 (4)		

Symmetry codes: (i) −*x*+1, −*y*, −*z*+2; (ii) *x*, *y*+1, *z*; (iii) −*x*+1, −*y*−1, −*z*+2; (iv) −*x*+3/2, *y*+3/2, −*z*+3/2; (v) *x*−1/2, −*y*−1/2, *z*−1/2; (vi) *x*, *y*−1, *z*; (vii) −*x*+3/2, *y*+1/2, −*z*+3/2; (viii) *x*+1/2, −*y*+1/2, *z*+1/2; (ix) −*x*+1/2, *y*−1/2, −*z*+3/2; (x) −*x*+1/2, *y*+1/2, −*z*+3/2; (xi) *x*+1/2, −*y*−1/2, *z*+1/2; (xii) −*x*+3/2, *y*−3/2, −*z*+3/2; (xiii) −*x*+3/2, *y*−1/2, −*z*+3/2; (xiv) *x*−1/2, −*y*+1/2, *z*−1/2.

### Hydrogen-bond geometry (Å, º)

**Table:**

*D*—H···*A*	*D*—H	H···*A*	*D*···*A*	*D*—H···*A*
C7—H7···O1	0.93	2.50	2.822 (3)	100
